# Combined Endoscope assisted Procedures (CEaP) as a complete treatment for neovascular glaucoma

**DOI:** 10.1371/journal.pone.0234798

**Published:** 2020-06-17

**Authors:** Yuan-Shao Cheng, Shih-Huan Lin, Chia-Jen Chang

**Affiliations:** 1 Department of Ophthalmology, Taichung Veterans General Hospital, Taichung, Taiwan; 2 Institute of Genomics and Bioinformatics, National Chung Hsing University, Taichung, Taiwan; 3 Department of Optometry, Central Taiwan University of Science and Technology, Taichung, Taiwan; Bascom Palmer Eye Institute, UNITED STATES

## Abstract

**Purpose:**

To investigate the effect and complications of Combined Endoscope assisted Procedures (CEaP): endoscopic cyclophotocoagulation and pars plana ablation (ECP-plus), along with endoscopic panretinal photocoagulation (PRP).

**Patients and methods:**

The study design is a retrospective and noncomparative interventional case series from a tertiary referral center in Taiwan. Patients experiencing vessel growth at the iris and anterior chamber angle, along with an IOP > 21 mmHg were included.

**Results:**

Twenty-five eyes from 23 patients were included over a 24-month period. After the procedures, all of them had a lower IOP value than their preoperative value. The mean IOP was 38.2± 7.1 mm Hg preoperatively, and 10.2± 4.7 mmHg (1 day), 13.8± 4.6 mmHg (1 week), 15.0± 5.3 mmHg (2 weeks), 17.4± 4.7 mmHg (1 month), 16.6± 4.1 mmHg (3 months), 16.0± 5.0 mmHg (6 months), and 15.7± 5.5 mmHg (12 months) postoperatively. At the 6th and 12th months, the IOP stabilized rate was 84% and 75%, respectively. Complications in the initial postoperative period (< 3 months) included uveitis (24%), and hyphema (16%), which were both resolved in the early postoperative period. Complications beyond 6 months included hypotony and phthisis bulbi in two patients (8%) in our study. There was no subject who suffered from retinal detachment, endophthalmitis or any other severe complications.

**Conclusions:**

The results of this study show that CEaP offers positive results in IOP lowering and NV regression. Additionally, CEaP is a complete treatment for NVG in controlling IOP and NV growth. The IOP lowering effects can be sustained upon completion of the treatment.

## Introduction

Neovascular Glaucoma (NVG), a severe form of secondary glaucoma, has been both a medical and surgical challenge for ophthalmologists for a long period of time. It is characterized by vessel proliferation involving the iris and the anterior chamber angle, with eventual angle closure and intractable elevation of Intraocular Pressure (IOP). The diagnosis requires details on the ocular and systemic past history of the patient, along with a complete ophthalmological examination. Common causes that lead to NVG are ischemic retinal conditions, such as Proliferative Diabetic Retinopathy (PDR), Branch Retinal Vein Occlusion (BRVO), Central Retinal Vein Occlusion (CRVO), Central Retinal Artery Occlusion (CRAO), and carotid artery occlusion. [1–3] Treatment choices for NVG include Panretinal Photocoagulation (PRP), filtering surgery, Anterior Retinal Cryotherapy (ARC), injection of Anti–Vascular Endothelial Growth Factor (Anti-VEGF), Cyclocryotherapy (CCT) and Transscleral Cyclophotocoagulation (TSCPC). [4] In addition to reducing IOP, it is important to note that NVG treatment should possess the ability to prevent further vessel proliferation. [5]

Procedures such as CCT and TSCPC can lower intraocular pressure in NVG by reducing aqueous humor formation, however they both carry a significant risk of complications, including marked inflammation, hypotony and, phthisis. [6, 7] With a single probe combining a diode endolaser, aiming beam, light source, and endoscope, a cyclodestructive procedure called an Endoscopic Cyclophotocoagulation (ECP) uses a laser which can be delivered to the target tissue under direct visualization at appropriate energy levels, which in turn helps ECP reduce IOP, and subsequently avoid severe tissue damage and inflammation. [8]

Endoscopic cyclophotocoagulation and pars plana ablation (ECP-plus) has been reported as an effective and safe treatment towards the relief of refractory glaucoma. [9] ECP-plus has shown positive IOP controlling results, with no additional significant side effects compared to those produced by ECP. We have devised a combination therapy, known as Combined Endoscope assisted Procedures (CEaP), which involves endoscopic cyclophotocoagulation and pars plana ablation (ECP-plus), combined with endoscopic panretinal photocoagulation. Here, we report on the efficacy and safety of CEaP in the treatment of neovascular glaucoma.

## Patients and methods

A retrospective study was conducted by collecting the clinical data of subjects visiting a tertiary referral center, the Taichung Veterans General Hospital, Taichung, Taiwan during the period from 01 November 2014 through 01 November 2018. This study was approved by the ethical research committee of Taichung Veterans General Hospital. Oral consent for use of clinical records was taken from patients during follow-up. Written consent was not acquired because it is a retrospective study. All patients’ information were anonymized prior to analysis. All research conformed to the tenets of the Declaration of Helsinki.

The inclusion criteria was comprised of those patients who were diagnosed with NVG, and whose treatment had failed with previous anti-glaucoma drugs, PRP, and anti-VEF treatment. Diagnosis of NVG was confirmed if the patient had vessel growth at the iris and an anterior chamber angle with an IOP > 21 mmHg. Failure following previous treatment was defined as the presentation of Neovascularization (NV) at the iris and the anterior chamber angle, along with an IOP > 21mmHg after treatment of more than 3 months.

The exclusion criteria were eyes with a Visual Acuity (VA) worse than one’s Hand Motion (HM), along with those patients who had received an intravitreal injection of anti-VEGF within 6 weeks prior to CEaP. Patients who had not received a lens extraction, together with those patients who had received glaucoma filtering surgery, glaucoma implants, ARC, CCT, or TSCPC were also excluded.

Patient’s past ocular, medical and surgical history was recorded. All of the subjects received both preoperative and postoperative IOP, VA, slit lamp, gonioscopy, and fundus examinations. IOP was determined by Goldmann applanation tonometry performed by an ophthalmologist. VA was tested using a tumbling E chart. We attempted to reduce the bias of IOP by averaging the measurements prior to surgery. Baseline IOP was defined as being the average of the two consecutive visits before surgery. Postoperative IOP was recorded at 1 day, 1 week, 2 weeks, 1 month, 3 months, 6 months, and 12 months. VA at either 6 or 12 months after the procedures (final follow-up) was recorded as the postoperative VA. Postoperative complications including hypotony (IOP<5 mm Hg for more than 2 weeks), severe uveitis associated with fibrin formation, corneal edema, hyphema, endophthalmitis, and retinal detachment were recorded if they were experienced by the patient. Stabilized IOP was defined as an IOP≤21 mm Hg, and lowered to a level greater than 20% from preoperative IOP, with the exclusion of phthisis bulbi.

All of the subjects received CEaP from the same surgeon. The endoscope system used was Endo Optiks E4 (Endo Optiks, Inc., Little Silver, NJ, USA). At the initial point of treatment, a standard 25-gauge 3 ports pars plana vitrectomy was performed, followed by a complete endoscopic PRP. Before the surgery, all patients have received PRP in outpatient clinic. During the endoscopic PRP, a supplementary 360-degree PRP was performed from equator to ora serrata. The distance between the endoscopic probe and the retina was 3–5 mm. Laser was shot with power of 120 to 150 mW, and duration of 0.15 to 0.2 seconds. The laser spots were applied as many as possible to cover at least one third of the retinal area except macula. After vitrectomy and endoscopic PRP, a two ports ECP-plus was performed to more than 270 degrees of the ciliary body. The coagulation time was set to continuous, and the energy was 150mw for ECP-plus. All of the subjects had a follow-up period of more than 6 months postoperatively. Antiglaucoma drops were prescribed if the IOP was found to be >21mmHg during any visit.

Statistical analyses were performed using the Statistical Package for the Social Science (IBM SPSS version 22.0; International Business Machines Corp, New York, USA). Pre-operative IOP was compared with post-operative follow-up IOP values using Friedman test and Dunn-Bonferroni post-hoc analysis. The cumulative successful rate was assessed according to the Kaplan-Meier analysis. A p-value of < 0.05 was considered statistically significant.

## Results

Twenty-five eyes from 23 subjects were included for the study over a 24-month period. Males and females were equally distributed in patient numbers. Eight had Proliferative Diabetic Retinopathy (PDR), eleven had Branch Retinal Vein Occlusion (BRVO), while four were diagnosed with Central Retinal Vein Occlusion (CRVO) (Table 1). The mean age amongst the patients was 61.9 ± 13.2 years. Preoperative VA ranged from No Light Perception (NLP) to 6/10. Preoperative IOP ranged from 27 to 52 mmHg (Table 2).

**Table 1:** Summary of characteristics of patients.

	N %
No. of patients/eyes	23 / 25
Gender (%)	
Male	12 (52.2%)
Female	11 (47.8%)
Age(yrs), mean ± SD	61.91 ±13.17
Disease (%)	
PDR	8 (34.8%)
CRVO	4 (17.4%)
BRVO	11 (47.8%)

PDR = proliferative diabetic retinopathy; CRVO = central retinal vein occlusion;

BRVO = branch retinal vein occlusion; SD = standard deviation.

**Table 2:** Summary of patient characteristics and their IOP values before and after operation.

Subject	Preoperative			Postoperative										
	VA	IOP	NV Iris/ angle	IOP 1 D	IOP 1 W	IOP 2 W	IOP 1 M	IOP 3 M	IOP 6 M	IOP 12M	VA 6M	VA 12M	NV (12M) Iris/ angle	Complications
1	6/60	35	-/+	10	12	14	22	20	18	18	6/60	6/60	-/-	-
2	6/15	29	+/+	9	16	14	25	22	24	22	6/20	6/30	-/+	Uveitis(1W,3+*)
3	HM	30	+/+	11	13	17	10	12	5	3	LP	NLP	N/A	Phthisis(6M)
4	6/30	47	+/+	13	22	18	24	16	14	N/A	6/30	N/A	-/-(6M)	-
5	3/60	48	-/+	22	25	21	23	19	18	18	6/60	6/60	-/+	-
6	3/60	36	+/+	9	19	16	17	20	22	22	1/60	1/60	+/+	Uveitis(1W,3+*)
7	6/20	29	+/+	6	10	14	19	15	12	12	6/15	6/20	-/-	-
8	6/10	36	-/+	5	11	18	26	23	21	N/A	6/12	N/A	-/+(6M)	-
9	HM	40	-/+	8	9	10	15	15	18	18	HM	HM	-/-	-
10-R	6/15	27	+/+	12	12	14	18	16	16	16	6/15	6/15	-/-	-
10-L	CF	42	+/+	15	14	16	23	21	18	18	1/60	CF	-/+	Hyphema(1W)
11	6/12	52	-/+	10	12	12	14	16	17	19	6/20	6/30	-/+	
12	HM	45	+/+	9	11	16	9	8	5	4	LP	NLP	N/A	Uveitis(1W,3+*) Hyphema(3M) Phthisis(1Y)
13	6/15	45	-/+	11	13	16	16	13	13	16	6/15	6/15	-/-	
14	3/60	37	+/+	3	16	16	18	13	13	15	1/60	1/60	-/-	
15	6/60	29	-/+	22	20	24	21	26	26	N/A	2/60	N/A	-/+(6M)	
16	2/60	35	-/+	9	12	12	15	16	16	N/A	6/60	N/A	-/-(6M)	
17	CF	44	+/+	6	10	7	17	9	9	13	HM	HM	+/+	Hyphema(1M)
18	6/15	39	-/+	5	11	11	13	15	15	16	6/12	6/12	-/-	
19	2/60	35	-/+	8	9	12	12	16	16	21	3/60	1/60	+/+	Uveitis(1W,2+*)
20-R	NLP	44	+/+	12	12	12	15	15	15	N/A	NLP	N/A	+/+(6M)	Hyphema(2W)
20-L	6/60	29	+/+	15	14	22	19	19	19	N/A	6/20	N/A	-/-(6M)	Uveitis(1W,3+*)
21	3/60	45	-/+	9	18	9	15	15	15	N/A	3/60	N/A	-/-(6M)	
22	6/20	44	+/+	4	5	5	11	16	16	N/A	6/20	N/A	-/+(6M)	
23	6/60	33	+/+	12	19	29	17	19	19	N/A	6/60	N/A	-/-(6M)	Uveitis(1W,3+*)

VA = visual acuity; IOP = intraocular pressure; NV = neovascularization; D = day; W = week; M = month; PDR = proliferative diabetic retinopathy; CRVO = central retinal vein occlusion; BRVO = branch retinal vein occlusion; HM = hand motion; CF = counting fingers; LP = light perception; NLP = no light perception.

* Standardization of Uveitis Nomenclature

**Fig 1:**
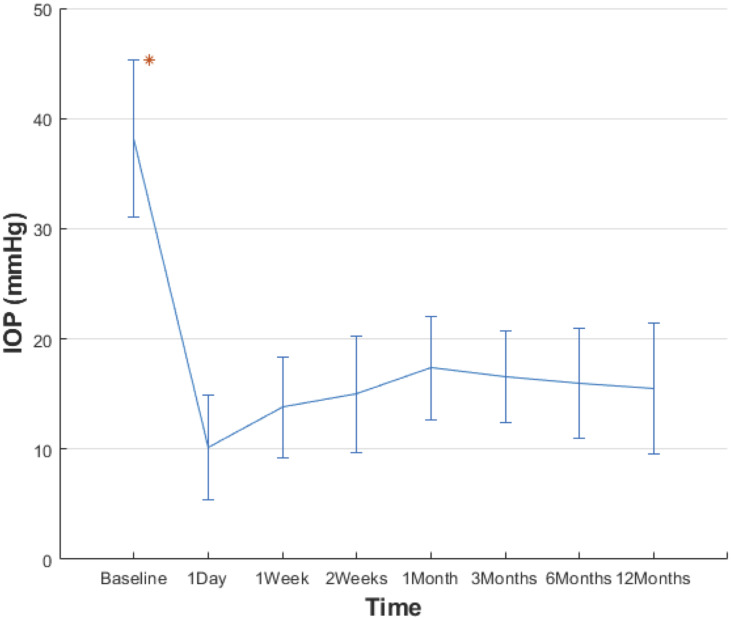
Mean IOP values before and after operation. *The error bars represent one standard deviation.

The IOP values measured over the study period are shown in Table 2. After the procedure, all patients’ IOP dropped below their preoperative values. Mean IOP was 38.2± 7.1 mm Hg prior to surgery. After the operation, mean IOP values measured at various times were: 10.2± 4.7 mmHg (1 day), 13.8± 4.6 mmHg (1 week), 15.0±5.3 mmHg (2 weeks), 17.4± 4.7 mmHg (1 month), 16.6± 4.1 mmHg (3 months), 16.0± 5.0 mmHg (6 months), and 15.7 ± 5.5 mmHg (12 months). The patients’ mean IOP values dropped after the procedure, and the effect can be sustained for 12 months. (Fig 1). All the IOP values (before and after operation) were compared using the Friedman test. The result showed statistical significancy (P<0.001). The post-hoc analysis (Dunn-Bonferroni) were used to further test the differences between baseline and every postoperative IOP values. The results show every postoperative IOP value is different to the baseline IOP (Table 3). Three of the patient’s values were >21mmHg despite treatment with anti-glaucoma drugs at the 6th month, with only two valued at >21mmHg at the 12th month. None of the patients received an intravitreal injection of anti-VEGF due to uncontrolled IOP during the follow-up period, or had prominent NV at the iris and the anterior chamber angle.

**Table 3:** Friedman test and the post-hoc analysis of the IOP values before and after operation.

	IOP (n = 25)		
	Median	IQR	*P* value
Baseline	37.0	(31.5–44.5)	<0.001***
1D	9.0	(7.0–12.0)	
1W	12.0	(11.0–17.0)	
2W	14.0	(12.0–17.5)	
1M	17.0	(14.5–21.5)	
3M	16.0	(15.0–19.5)	
6M	16.0	(13.5–18.5)	
Post-hoc analysis (Dunn-Bonferroni)	Baseline vs 1D		<0.001***
	Baseline vs 1W		<0.001***
	Baseline vs 2W		<0.001***
	Baseline vs 1M		0.001**
	Baseline vs 3M		<0.001***
	Baseline vs 6M		<0.001***

IQR = interquartile range; D = day; W = week; M = month

**p<0.01,

***p<0.001

Based on the clinical experience of the surgeon, we used post-op 6-months as the date for VA assessment. At post-op 6 months, the VA was stable or improved in 16 of the 25 eyes (64%). We also assessed the VA for those who had follow-up at 12-months after the surgery and it was stable or improved in 8 of the 16 eyes (50%). Twelve of the involved eyes had total NV regression, with the other 3 showing partial regression. No intraoperative complications were found after surgery, though three patients developed uveitis or hyphema. Six of the 25 eyes (24%) experienced uveitis with fibrin formation at the anterior chamber, with the condition being resolved within 3 months. Four of the 25 eyes (16%) had postoperative hyphema, a condition which was also resolved within 3 months. One eye each of CRVO and of BRVO, which had received 360-degree ECP-plus, showed a satisfactory drop in IOP during the initial postoperative period, however this later transformed into hypotony and phthisis bulbi at the 6th and 12th months, respectively.

**Fig 2:**
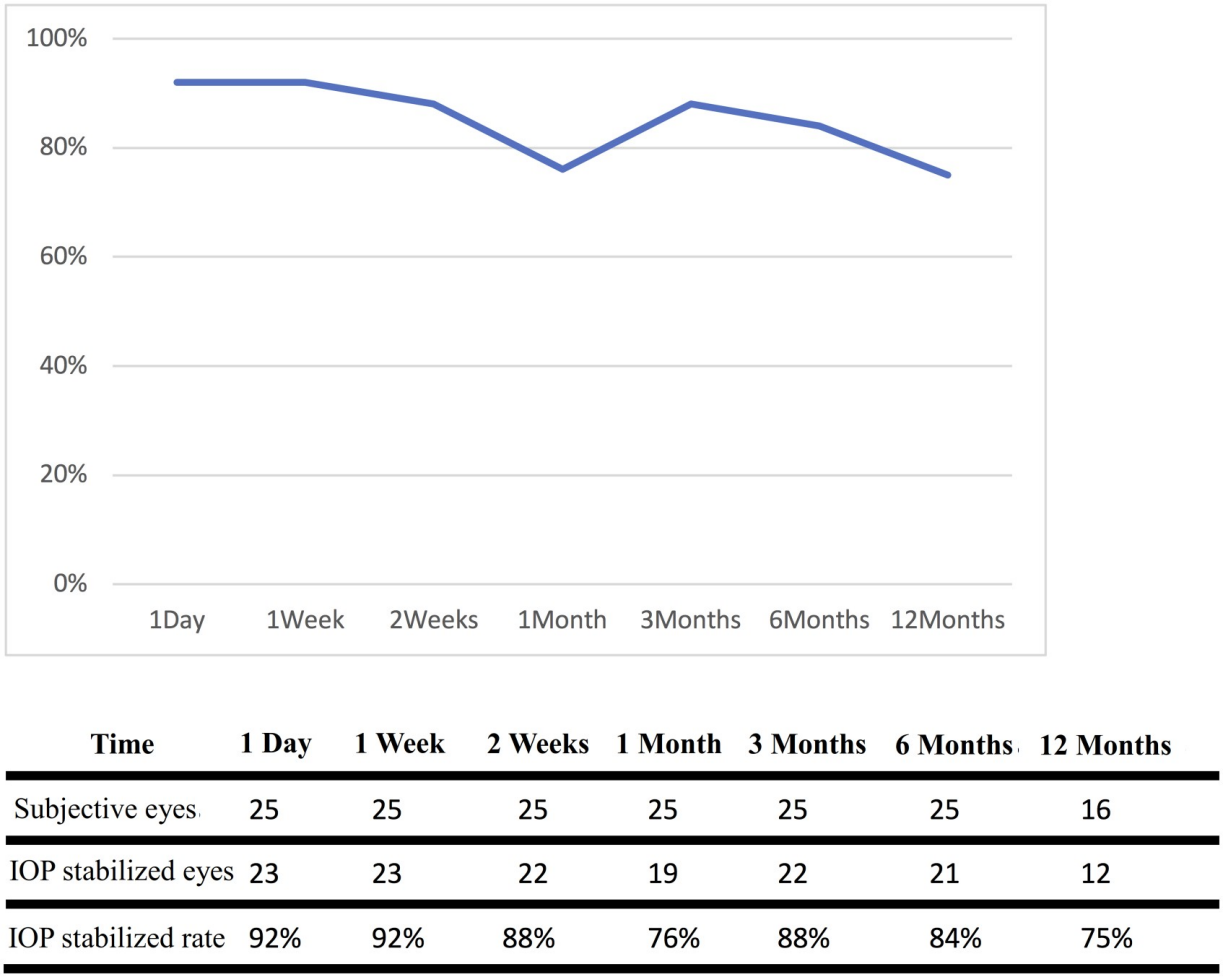
IOP stabilized rates of operation at different post-operative time.

**Fig 3:**
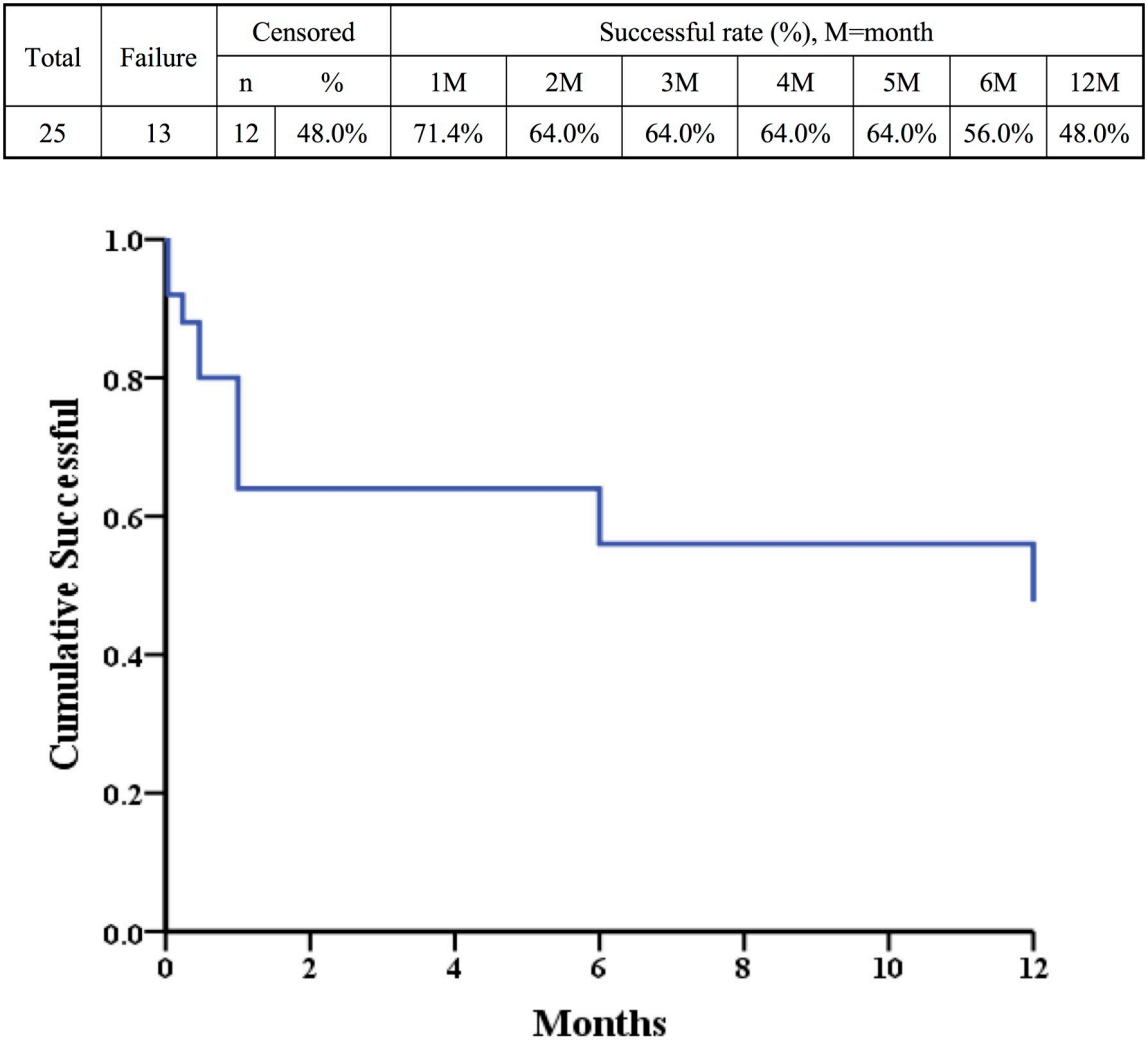
The Kaplan-Meier analysis for assessing the cumulative successful rate.

No subjects suffered from retinal detachment, endophthalmitis or any other severe complications. By our definition, IOP stabilized rate fell between 75%-92%. At the 6th and 12th months, the IOP stabilized rate was 84% and 75%, respectively (Fig 2). The Kaplan-Meier cumulative successful rate was shown in Fig 3.

## Discussion

There are two key aspects regarding the management of NVG. The first one is treatment of the ischemic state and neovascularization; and the other is treatment of elevated IOP once it has been established. When considering treatment for the ischemic state, Panretinal Photocoagulation (PRP) is a useful method, as it reduces the global retinal oxygen demand by destroying the nonessential ischemic retinal tissue, thus removing the stimulus for the production of vasoproliferative factors. However, PRP is difficult to perform in patients experiencing NVG with media opacities such as corneal edema and dense cataract. With the assistance of an endoscope, PRP may be performed in the presence of media opacities. Furthermore, through the pars plana approach, the laser could be applied up to the ora serrata more comprehensively, as compared to the external approach performed in a clinic.

Another current and popular treatment for neovascularization regression involves the injection of anti-VEGF. This approach is expected to directly decrease the level of intraocular VEGF, causing the regression of neovascularization in the retina, anterior chamber angle, and iris. [10] However, intraocular anti-VEGF is locally short-term retaining, generally between 4 to 6 weeks, rather than providing long-term inhibition of VEGF releasing. [11] The majority of patients required multiple injections, additional laser treatment, and IOP-lowing surgery. Anti-VEGF therapy is considered an effective adjuvant, rather an isolated treatment for neovascular glaucoma. Preoperative intravitreal injection of VEGF has been advocated to promote neovascularization regression, while increasing the surgical success rate in patients with NVG. When compared to anti-VEGF therapy, PRP radically improves retinal ischemia and thereby inhibits VEGF release, which should be a significant component in treatment for NVG. [12, 13] We aimed to identify the CEaP treatment effect without the confounding factor of Anti-VEGF injections. Therefore, patients who received any intravitreal injection of anti-VEGF within 6 weeks prior to CEaP were excluded, while none of the patients who received CEaP underwent any intravitreal injection of Anti-VEGF during the follow-up period in this study. Further studies are still necessary in order to explore the effect of when Anti-VEGF injections are combined with CEaP during NVG treatment.

Filtering surgery, including a trabeculectomy and tube shunt surgery using Glaucoma Drainage Devices (GDDs), is an effective method for the treatment of elevated IOP in the majority of glaucoma patients. However, the outcomes in the treatment of NVG are unsatisfactory. Hyphema, tube shunt exposure, hypotony or loss of vision are not rare occurrences amongst NVG patients after these procedures. [14, 15] NVG patients possess a highly inflammatory environment in the aqueous humor than patients who have another type of glaucoma, [16, 17] which may be related to the low long-term IOP stabilized rate caused by fibrous tissue obstruction or external scarring. Intra- and postoperative use of anti-metabolites has been proposed for bringing about a reduction in the failure rate. Previous studies have reported the effectiveness of filtering surgery in NVG patients. For instance, Yuji et al. reported a 62.6% IOP stabilized rate at 1 year after undergoing a trabeculectomy with mitomycin C, for patients experiencing NVG. [18] Yalvac et al. reported a 63.2% IOP stabilized rate at 1 year for patients after Ahmed glaucoma valve implantation. [19] In our study, the IOP stabilized rate was 75% at 1 year, which is not inferior to the above-mentioned studies, however longer-term follow-up data is still pending.

Recently, a growing number of investigations have offered promising results for ECP as a primary surgery in eyes with good vision. With the assistance of an endoscope, surgeons can better visualize anatomical structures that are not visible with optical microscopes, and thus avoid potential overtreatment and surrounding tissues damage. ECP of the ciliary processes through use of an anterior chamber approach has been reported to reduce IOP by approximately 30%. [20, 21] The ECP-plus, a modified ECP procedure, has been reported as a laser application to the anterior and posterior ciliary processes and pars plana by the pars plana approach. ECP-plus photocoagulates any extension of the secretory ciliary epithelium from pars plicata onto pars plana, and may also increase uveoscleral outflow. [9] Because of its more aggressive nature, it is not surprising that ECP-plus induced more IOP lowering than other approaches. Feinstein et al. compared treatment outcomes between ECP-plus and anterior ECP. [22] Their results found that patients who underwent ECP-plus achieved a higher IOP stabilized rate at 2 years postoperatively (80% vs 33.3%, P < 0.001), along with a greater IOP decrease in the ECP-plus group when compared to the anterior ECP group (14.3 mmHg (52%) vs 5.2 mmHg (24%), P = 0.001).

The endoscopic laser is also helpful for PRP of the entire retina. In our study, the CEaP combined ECP-plus and endoscopic PRP, providing an approximately 60% IOP reduction during the postoperative 6- and 12- month periods when compared to baseline IOP. This value is much greater than those taken from reports involving ECP performed by the anterior chamber approach. The results in this study are compatible with previous data, which has suggested that pars plana ECP is more effective than anterior chamber approach ECP. Possible mechanisms for improved IOP reduction with pars plana approach ECP have been pointed out, and include direct visualization and treatment of the entire ciliary processes, and the intervening regions between processes and the pars plana. [23]

The long-term results of ECP have been reported in previous studies, showing that the ECP effect was sustained over at least 12 months. [24, 25] It has been reported that IOP was controlled to <21 mmHg for 1 year in 68% to 87% of 73 eyes which received ECP by the pars plana approach. [26] The IOP stabilized rate was still at 75% at the 12th month in our study. Since endoscope assisted PRP led to favorable NV regression, while inhibiting NVG by reducing retinal ischemia, the combination of ECP-plus and endoscope assisted PRP undoubtedly has a long effect.

The cumulative success rate at 1 year was 48% in our Kaplan-Meier survival curve, which is lower than other studies (78% by Tan et al. [9] and 85% by Feinstein et al. [22]). In Kaplan-Meier survival curve of these studies, short-term post-op IOP of 1 day, 1 week, and 2 weeks were not included. The main reason for less than 50% cumulative success rate at 1 year in our study was that we included short-term post-op IOP of 1 day, 1 week, and 2 weeks. The short-term post-op IOP had obvious fluctuations especially in the first post-op month, then the IOP became more and more stable. These fluctuations of IOP in the first post-op month were classified as failure groups and lowered the cumulative success rate in the Kaplan-Meier survival curve. As a result, the cumulative success rate declined 30% soon at the first post-op month.

A study regarding the use of a less aggressive laser of the posterior ciliary processes (210-degrees) had a mean IOP reduction of 37% at 12 months. [21] In another study with full 360-degree ciliary ablation, the mean IOP was reduced approximately 70% at 1 year postoperatively. [27] The mean IOP reduction was approximately 60% at both 6 and 12 months in our study (more than 270-degrees). These results show that the magnitude of IOP reduction seems to be related to the extent of the treatment area. This is not surprising because the more extensive photocoagulation there is of the secretory ciliary epithelium, the more complete reduction in aqueous humor production. However, additional larger comparative studies are still necessary in order to support this hypothesis.

The destructive nature of ECP causes physicians to become concerned about the complications which occur. In a study regarding pars plana ECP, complications included hypotony, choroidal effusion, fibrinous anterior chamber reaction, and choroidal hemorrhage. [21] In our study, the early complications of ECP and endoscope assisted PRP were uveitis (24%), and hyphema (16%), which were both resolved in the early postoperative period. The complication profiles were similar to that of another study regarding anterior approach ECP, and included hypotony or choroidal effusion (4%), fibrinous uveitis (24%), hyphema (12%), and cystoid macular edema (10%). [28] In our study, late complications included hypotony and phthisis bulbi, which occurred during the late postoperative period (after 6 months) in two patients (8%). Similarly, in studies by Tan et al. [9] (pars plana ECP, received 300 to 330-degree ciliary ablation) and Marra et al. [27] (pars plana ECP, received 360-degree ciliary ablation), both had an 8% complication rate for prolong hypotony. These results indicate that the combined procedure, ECP and endoscope assisted PRP, offer equal safety to that of the anterior approach ECP and pars plana ECP.

CEaP is both safe and effective, but also has certain limitations, including its cost and technical feasibility. The ophthalmic endoscope system is expensive. However, after the initial capital outlay for the ECP laser console, with repeat and successive use, the unit cost per procedure will continue to decrease. [29] Another concern is that proficiency in endoscopy requires the skills which have a relatively steep learning curve for operators.

In this study, the IOP lowering effects can be sustained for 6–12 months after treatment. The CEaP offered positive results in both IOP lowering and NV regression, offering both durable effects and safety for neovascular glaucoma. However, there were some limitations in our study, including its retrospective nature, relative small case number, and lack of a control group. Further research with a larger sample size and longer follow-up period is still required in order to confirm our findings.

## Supporting information

S1 Table Combined Endoscope assisted Procedures (CEaP) as a complete treatment for neovascular glaucoma. (DOCX)
