# Supplementary material for: Combined Endoscope assisted Procedures (CEaP) as a complete treatment for neovascular glaucoma
Source: PLoS One. 2020 Jun 17;15(6):e0234798. doi: 10.1371/journal.pone.0234798 (PMC7299373; doi:10.1371/journal.pone.0234798)
Supplement: S1 Table — (DOCX) [file pone.0234798.s001.docx]

| Subject | Preoperative | | | Postoperative | | | | | | | | | | |
| --- | --- | --- | --- | --- | --- | --- | --- | --- | --- | --- | --- | --- | --- | --- |
|  | VA | IOP | NV Iris/ angle | IOP  1 D | IOP  1 W | IOP  2 W | IOP  1 M | IOP  3 M | IOP  6 M | IOP  12M | VA  6M | VA  12M | NV (12M)  Iris/ angle | Complications |
| 1 | 6/60 | 35 | -/+ | 10 | 12 | 14 | 22 | 20 | 18 | 18 | 6/60 | 6/60 | -/- | - |
| 2 | 6/15 | 29 | +/+ | 9 | 16 | 14 | 25 | 22 | 24 | 22 | 6/20 | 6/30 | -/+ | Uveitis(1W,3+^*^) |
| 3 | HM | 30 | +/+ | 11 | 13 | 17 | 10 | 12 | 5 | 3 | LP | NLP | N/A | Phthisis(6M) |
| 4 | 6/30 | 47 | +/+ | 13 | 22 | 18 | 24 | 16 | 14 | N/A | 6/30 | N/A | -/-(6M) | - |
| 5 | 3/60 | 48 | -/+ | 22 | 25 | 21 | 23 | 19 | 18 | 18 | 6/60 | 6/60 | -/+ | - |
| 6 | 3/60 | 36 | +/+ | 9 | 19 | 16 | 17 | 20 | 22 | 22 | 1/60 | 1/60 | +/+ | Uveitis(1W,3+^*^) |
| 7 | 6/20 | 29 | +/+ | 6 | 10 | 14 | 19 | 15 | 12 | 12 | 6/15 | 6/20 | -/- | - |
| 8 | 6/10 | 36 | -/+ | 5 | 11 | 18 | 26 | 23 | 21 | N/A | 6/12 | N/A | -/+(6M) | - |
| 9 | HM | 40 | -/+ | 8 | 9 | 10 | 15 | 15 | 18 | 18 | HM | HM | -/- | - |
| 10-R | 6/15 | 27 | +/+ | 12 | 12 | 14 | 18 | 16 | 16 | 16 | 6/15 | 6/15 | -/- | - |
| 10-L | CF | 42 | +/+ | 15 | 14 | 16 | 23 | 21 | 18 | 18 | 1/60 | CF | -/+ | Hyphema(1W) |
| 11 | 6/12 | 52 | -/+ | 10 | 12 | 12 | 14 | 16 | 17 | 19 | 6/20 | 6/30 | -/+ |  |
| 12 | HM | 45 | +/+ | 9 | 11 | 16 | 9 | 8 | 5 | 4 | LP | NLP | N/A | Uveitis(1W,3+^*^)  Hyphema(3M)  Phthisis(1Y) |
| 13 | 6/15 | 45 | -/+ | 11 | 13 | 16 | 16 | 13 | 13 | 16 | 6/15 | 6/15 | -/- |  |
| 14 | 3/60 | 37 | +/+ | 3 | 16 | 16 | 18 | 13 | 13 | 15 | 1/60 | 1/60 | -/- |  |
| 15 | 6/60 | 29 | -/+ | 22 | 20 | 24 | 21 | 26 | 26 | N/A | 2/60 | N/A | -/+(6M) |  |
| 16 | 2/60 | 35 | -/+ | 9 | 12 | 12 | 15 | 16 | 16 | N/A | 6/60 | N/A | -/-(6M) |  |
| 17 | CF | 44 | +/+ | 6 | 10 | 7 | 17 | 9 | 9 | 13 | HM | HM | +/+ | Hyphema(1M) |
| 18 | 6/15 | 39 | -/+ | 5 | 11 | 11 | 13 | 15 | 15 | 16 | 6/12 | 6/12 | -/- |  |
| 19 | 2/60 | 35 | -/+ | 8 | 9 | 12 | 12 | 16 | 16 | 21 | 3/60 | 1/60 | +/+ | Uveitis(1W,2+^*^) |
| 20-R | NLP | 44 | +/+ | 12 | 12 | 12 | 15 | 15 | 15 | N/A | NLP | N/A | +/+(6M) | Hyphema(2W) |
| 20-L | 6/60 | 29 | +/+ | 15 | 14 | 22 | 19 | 19 | 19 | N/A | 6/20 | N/A | -/-(6M) | Uveitis(1W,3+^*^) |
| 21 | 3/60 | 45 | -/+ | 9 | 18 | 9 | 15 | 15 | 15 | N/A | 3/60 | N/A | -/-(6M) |  |
| 22 | 6/20 | 44 | +/+ | 4 | 5 | 5 | 11 | 16 | 16 | N/A | 6/20 | N/A | -/+(6M) |  |
| 23 | 6/60 | 33 | +/+ | 12 | 19 | 29 | 17 | 19 | 19 | N/A | 6/60 | N/A | -/-(6M) | Uveitis(1W,3+^*^) |
